# Effect of a Recycled Phosphogypsum Modifier on the Performance of High-Content Phosphogypsum Cementing Materials

**DOI:** 10.3390/ma18122807

**Published:** 2025-06-14

**Authors:** Jiuyang Lian, Chiqiu Wu, Zhonghe Shui, Wei Lyu

**Affiliations:** 1 School of Materials Science and Engineering, Wuhan University of Technology, Wuhan 430070, China; lianjoy@whut.edu.cn; 2 Hubei Changyao New Materials Co., Ltd., Yichang 443000, China; cydg@vip.163.com (C.W.); 15871585473@139.com (W.L.); 3 State Key Laboratory of Silicate Materials for Architectures, Wuhan University of Technology, Wuhan 430070, China

**Keywords:** high-content phosphogypsum, dehydration phases, crystal seed, setting time, early strength

## Abstract

Phosphogypsum, a byproduct of phosphate fertilizer production, represents a significant environmental concern due to its large-scale production and low utilization rates. Although preparing phosphogypsum-based cementitious materials offers a potential solution to these issues, high-content phosphogypsum cementitious systems encounter significant technical barriers, including long setting durations and insufficient early-age strength development, thereby restricting their practical implementation. Hence, this research developed innovative modifiers through an environmentally friendly low-temperature thermal activation process (100–160 °C) utilizing recycled phosphogypsum aggregates and circumventing the substantial carbon emissions associated with conventional modification approaches. Systematic characterization demonstrated that the dehydration phase modifier synthesized at 120 °C (DH120) exhibited optimal phase composition, resulting in a 35.7% enhancement in its 14-d compressive strength (9.8 MPa vs. 7.2 MPa for the control) and an 11.3% reduction in its initial setting time (27.5 vs. 31.0 h for the control). Microstructural characterization by low-field nuclear magnetic resonance and X-ray diffractometry revealed that DH120 effectively enhanced refinement of the pore structure (37.7% mesopore volume reduction) and promoted the ettringite crystallization kinetics. This work establishes a sustainable framework for utilizing industrial byproducts in cementitious material systems.

## 1. Introduction

The continual development of phosphogypsum hydraulic cementitious materials, such as gypsum slag cement and supersulfated slag cement, has led to progressive increases in the content of phosphogypsum over the years [1,2,3,4,5]. To further enhance phosphogypsum utilization and mitigate the environmental and land resource pressures caused by its stockpiling, high-content phosphogypsum cementitious (HCPC) materials, which typically contain phosphogypsum contents exceeding 80%, have been developed [6,7]. However, owing to the limited content of active cementitious materials in this system (e.g., slag powder), chemical reactions primarily occur on the surfaces of the phosphogypsum particles, resulting in the insufficient formation of hydration products, leading to challenges such as low early strengths, high porosities, low softening coefficients, and prolonged setting times. More specifically, this slag exhibits low reactivity, resulting in the insufficient formation of early hydration products; consequently, it is necessary to incorporate modifiers to promote the reaction.

Previous studies have demonstrated that seed modifiers, such as ettringite seeds and C-S-H seeds, can reduce the Gibbs free energy of crystal nucleation, thereby promoting the early formation of ettringite and C-S-H gels and enhancing the overall hydration process [8,9,10,11]. For instance, Li et al. [12] demonstrated that incorporating 7% ettringite seeds in supersulfated phosphogypsum slag cement systems accelerates hydration; induces the rapid formation of ettringite in early-stage pores; and improves the matrix strength, setting time, and pore structure. Similarly, Zhang et al. [13] found that recycled concrete fines enhance hydration product growth by providing nucleation sites, thereby increasing the hydration degree and ultimately strengthening the cementitious matrix.

The thermal activation of hydration products at different temperatures yields dehydration phases with advantageous properties, such as shortened setting times and rapid rehydration rates. For example, Zhai et al. [14] observed that the incorporation of dehydrated cement paste can effectively reduce the setting time and enhance the strength of ordinary Portland cement. In addition, Sun et al. [15] demonstrated that introducing dehydrated C-S-H gel phases, aluminum phases, and calcium hydroxide into supersulfated phosphogypsum systems shortens the setting time and promotes early strength development. However, these methods typically require high-temperature treatment, which increases production costs and carbon emissions, thereby conflicting with low-carbon environmental principles. In high-content phosphogypsum cementitious materials, the main hydration products decompose at specific temperatures, namely, 90–100 °C for ettringite, 200–800 °C for C-S-H gel, and 100–200 °C for unreacted phosphogypsum [16,17,18,19].

To overcome these limitations, this study focuses on the development of an optimized low-temperature thermal activation process (≤160 °C) for producing modifiers from recycled phosphogypsum aggregates and systematically investigates the synergistic mechanisms between the seed and dehydration phase modifiers in terms of regulating the hydration kinetics and microstructural development. Through a combination of mechanical and thermal activation techniques, phosphogypsum slag aggregate waste is transformed into seed modifiers and dehydration phase modifiers, respectively. Subsequently, the influences of these modifiers on the setting behaviors, hardening characteristics, and mechanical performances of high-content phosphogypsum cementitious systems are comprehensively evaluated to establish a scientific basis for their practical implementation in such material systems.

## 2. Materials and Methods

### 2.1. Materials

The phosphogypsum used in this study was obtained from the Dongxiquan phosphogypsum slag yard of Zhongfu Chemicals (Yichang, Hubei Province, China). The P.O 42.5 Ordinary Portland Cement was sourced from Huaxin Cement Co., Ltd. (Wuhan, Hubei Province, China), and the ground granulated blast furnace slag powder (GGBFS) was provided by Lingshou County Dali Mineral Products Co., Ltd., Shijiazhuang, China. The recycled fine aggregate powder was prepared from phosphogypsum slag aggregate waste (phosphogypsum/slag powder/cement = 88:6:6 wt%) produced by a local enterprise (Hubei Linlong New Material Technology Co., Ltd., Yichang, Hubei Province, China). The waste was processed by drying at 50 °C until reaching a constant weight, and then by grinding and sieving (200-mesh square hole sieve) to obtain construction waste recycled cementitious material powder (RCMP) with a particle size of less than 74 µm.

**Figure 1:**
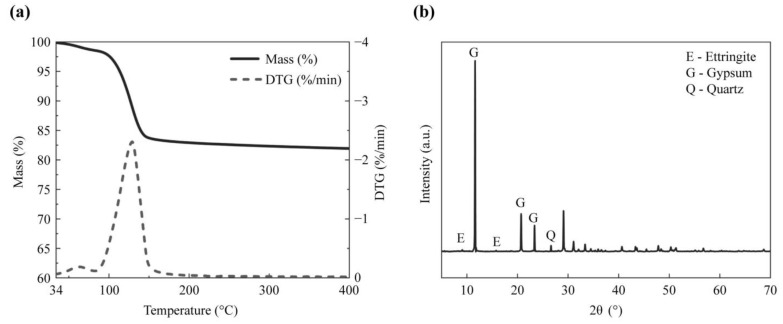
Analysis of the waste RCMP. (**a**) Thermogram, and (**b**) X-ray diffractogram.

The chemical compositions of all materials were analyzed by X-ray fluorescence spectrometry (XRF, PANalytical Zetium XRF). The chemical and mineral compositions of the RCMP were further characterized using X-ray diffractometry (XRD, D8 Advance) and thermal analysis (STA200 thermal analyzer, Hitachi, Japan). Detailed characterization results, including the key trends and findings, are presented in Table 1 and Figure 1.

### 2.2. Specimen Preparation

#### 2.2.1. Preparation of the Modifiers

The waste phosphogypsum-based aggregate was crushed and ground by the horizontal ball mill (GMS5-1, Changsha Miqi Instrument & Equipment Co., Ltd., Changsha, Hunan Province, China) with a ball/material ratio of 3:1 at 200 rpm for 1.5 h and then sieved through a 200-mesh sieve to obtain the waste RCMP, which served as a crystal seed (CS) modifier. The RCMP was thermally treated using an electric blast drying oven (101-00B Electrothermal constant temperature blast drying oven). The sample was heated at a rate of 5 °C/min. After reaching the predetermined temperature, the material was maintained at this temperature for 4 h, followed by natural cooling to 20–26 °C within the drying chamber. The final product was then sealed for storage, yielding the dehydration phase (DH) modifier, as detailed in Table 2.

#### 2.2.2. Characterization of the Modifiers

The particle size distributions of the CS and DH modifiers were analyzed using a Mastersizer 2000 laser diffraction analyzer (Malvern PANalytical, Malvern, UK), with isopropanol serving as the dispersion medium to ensure optimal particle separation. For phase composition analysis, XRD was performed on a Rigaku SmartLab SE diffractometer (Rigaku Corporation, Akishima, Tokyo, Japan) equipped with a Cu Kα radiation source. XRD was conducted under operational conditions of 40 kV and 40 mA, employing a continuous scanning mode from 2θ values of 5–70°. The scanning parameters were carefully optimized, giving a scan rate of 5°/min and a step size of 0.02°.

#### 2.2.3. Sample Preparation

The formulation parameters for the phosphogypsum-modified slag cementitious systems with equivalent substitution levels are presented in Table 3, wherein a constant water/binder (w/b) ratio of 4:10 was employed. The specimen fabrication protocol adhered to Chinese National Standard GB/T 17671-1999 [20]. For each mixing proportion outlined in the table, three cubic test specimens (40 × 40 × 40 mm^3^) were cast and cured under standardized conditions (20 ± 1 °C, 95% RH) until testing. The mechanical properties of the samples were subsequently evaluated after 7, 14, and 28 d of storage, and the compressive strengths are reported as the mean ± the standard deviation (SD).

Following the strength evaluations, the test specimens were broken and soaked in anhydrous ethanol for 24 h to prevent hydration reactions. Subsequently, the samples were dried at 40 °C for 24 h. A subset of the samples was allocated for additional analysis; one portion was examined using LF-NMR to evaluate its pore structure, while the other was retained for subsequent mineralogical and microstructural investigations.

### 2.3. Test Methods

#### 2.3.1. Workability Testing

The macroscopic physical properties of the paste, including the setting time and fluidity, were evaluated to assess its workability and hardening behavior. The initial and final setting times were determined in compliance with the Chinese national standard GB/T 1346-2011 [21], employing a standardized Vicat apparatus for precise measurement.

#### 2.3.2. Mechanical Performance Testing

The compressive strengths of the 40 × 40 × 40 mm^3^ cubic specimens were tested using a universal testing machine (DYE-300S Compression Tester, Wuxi Dejiayi Testing Instrument Co., Ltd., Wuxi, Jiangsu Province, China) according to the national standard GB/T 17671-1999 [20] under a loading rate of 0.5 kN/s.

The softening coefficient (*φ*), defined as the ratio of compressive strength in the water-saturated state (*f_sat_*) to that in the dry state (*f_dry_*), was calculated as follows to evaluate the water resistance:

$$\varphi =\frac{f_{dry}}{f_{sat}}.$$

Water-saturated specimens were prepared by immersion in deionized water for 48 h, while the dry specimens were obtained by vacuum drying at 40 ± 2 °C (vacuum ≤ 10 Pa) until reaching a constant weight (mass change <0.1%). The compressive strength was measured at a loading rate of 0.5 kN/s, and the softening coefficient was calculated from the mean values obtained from three parallel samples. A higher softening coefficient (i.e., closer to 1) indicates superior water resistance and modifier effectiveness.

#### 2.3.3. Hydration Heat

The hydration heat was assessed using a TAM Air C80 isothermal calorimeter (TA Instruments, New Castle, DE, USA), which exhibits a sensitivity of ±0.1 mW/g, following calibration with indium reference standards. The sample paste, prepared according to the conditions outlined in Table 3 (w/b ratio of 4:10), was placed in a sealed test cell, and the hydration heat release rate and cumulative heat release were monitored for 10 d at 25 ± 0.1 °C.

#### 2.3.4. Characterization of the Hydration Products

For phase analysis of the hydration products, specimens cured for 14 d were first crushed into granules and immediately immersed in anhydrous ethanol (purity ≥ 99.7%) for 24 h to terminate further hydration. After immersion, the samples were removed from ethanol, filtered, and vacuum-dried at 40 ± 2 °C for 24 h (vacuum ≤ 10 Pa). Subsequently, the dried granular samples were ground into fine powders and sieved through a 200-mesh sieve (aperture 75 µm) to obtain a homogeneous powder for further testing. More specifically, the crystal phase compositions of the hydration products were analyzed by XRD using a Rigaku SmartLab SE diffractometer (Tokyo, Japan) equipped with a Cu-Kα radiation source. Data acquisition was conducted over a 2θ range of 5–70° using a continuous scanning method, with a step size of 0.02° and a scan rate of 5°/min. Additionally, the thermal behavior of the cement hydration products was investigated using TG-DSC (HITACHI STA200, Tokyo, Japan). These experiments were conducted in a nitrogen environment, wherein the temperature was increased linearly from 30 to 400 °C at a heating rate of 10 °C/min.

#### 2.3.5. Microstructure Testing

The pore architectures of the 28-d-cured phosphogypsum-based cementitious composites were examined using LF-NMR (MesoMR12-060H-1 analyzer, Suzhou Niumag Analytical Instrument Co., Ltd., Suzhou, Jiangsu Province, China). The experimental configuration was established with the following optimized parameters: magnetic field strength = 0.5 ± 0.01 T, resonance frequency = 21 MHz, pulse sequence = CPMG (τ = 200 μs, NECH = 8000), temperature = 32.0 ± 0.5 °C, cumulative number of scans (NS) = 64, and relaxation waiting time (TW) = 2000. All samples were vacuum-saturated for 48 h before testing, with three-dimensional gradient coil compensation applied to minimize the magnetic field distortion (<50 ppm variation). The transverse relaxation time (*T*_2_) distributions were deconvoluted using the non-negative least squares (NNLS) algorithm, converting the *T*_2_ times to pore sizes via the following equation:

*r* = *C*⋅*T*_2_

where *C* = 3.05 μm/s (calibrated according to mesoporous silica standards).

Microstructural characterization was performed using field-emission scanning electron microscopy (FE-SEM, TESCAN MIRA LMS, Czech Republic). For this purpose, small specimen fragments were carefully mounted on conductive adhesive and sputter-coated with a thin gold layer (45 s at 10 mA) using a Quorum SC7620 sputter coater to ensure an appropriate surface conductivity. Finally, high-resolution SEM images were acquired at an optimized accelerating voltage of 3 kV.

## 3. Characterization of Modifiers

### 3.1. Phase Composition

**Figure 2:**
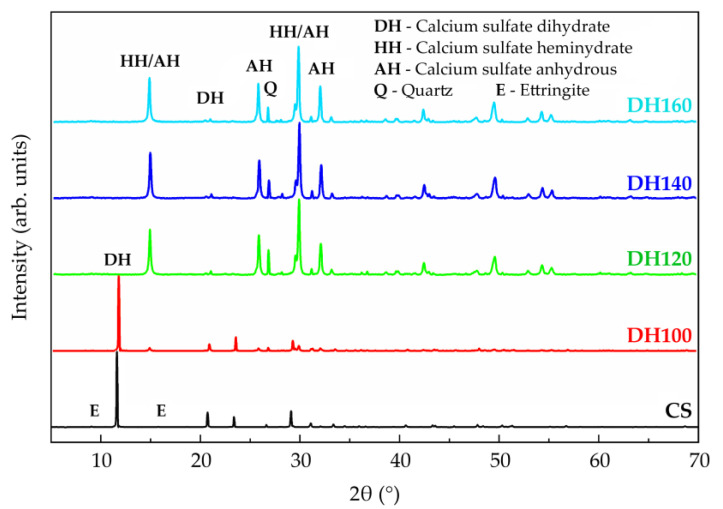
XRD patterns of the CS and DH series modifiers.

Figure 2 presents the phase composition of the crystal phase compositions of the CS and DH samples. The CS sample consists of gypsum, ettringite, and quartz phases. The DH100 sample contains gypsum, hemipelagic gypsum, and quartz phases. The DH120 sample consists of hemipelagic gypsum and quartz phases. Both the DH140 and DH160 samples contain hemipelagic gypsum, anhydrite gypsum, and quartz phases. At 100 °C, the ettringite component present in the DH100 sample decomposes to generate meta-ettringite [16], which is accompanied by the formation of hemihydrate gypsum, indicating partial dehydration of the dihydrate gypsum. At 120–140 °C, the dihydrate gypsum present in DH120 converts into hemihydrate gypsum, while in the DH140 sample, the hemihydrate gypsum component begins to transform into anhydrite. At 160 °C, the hemihydrate gypsum present in DH160 further dehydrates into the anhydrite form. These phase transformations are critical for enhancing the setting, hardening, and early strength development characteristics of the modified recycled cementitious material.

### 3.2. Particle Size Distribution

**Figure 3:**
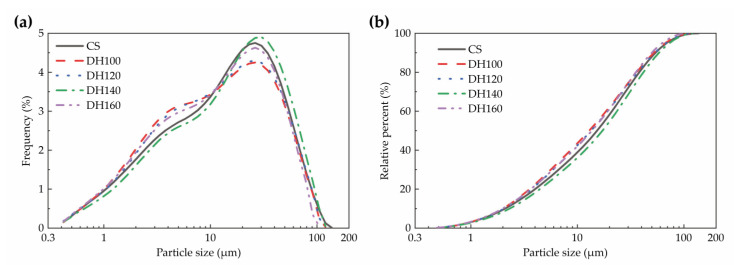
Particle size distributions of the modifier. (**a**) Particle size distribution curves, and (**b**) cumulative particle size distributions.

As illustrated in Figure 3 and Table 4, the thermal treatment temperature affected the particle size characteristics of the modifiers. The DH series samples (DH100-DH160) consistently showed finer particle sizes than the CS sample, with D50 reductions of 12.0–15.5% (15.296 μm → 12.924–13.675 μm) and narrower size distributions (Span decreasing from 3.40 to 3.65–3.81). More specifically, heat treatment was found to effectively refine the fine particles of the waste aggregate powder, primarily through the phase transformation of dihydrate gypsum to hemihydrate gypsum, which has a looser structure and a higher specific surface area, thereby enhancing the ability of this material to adsorb free water [22]. Therefore, precise control of the heat treatment temperature is essential for optimizing the particle size and material performance.

## 4. Results and Discussion

### 4.1. Workability

**Figure 4:**
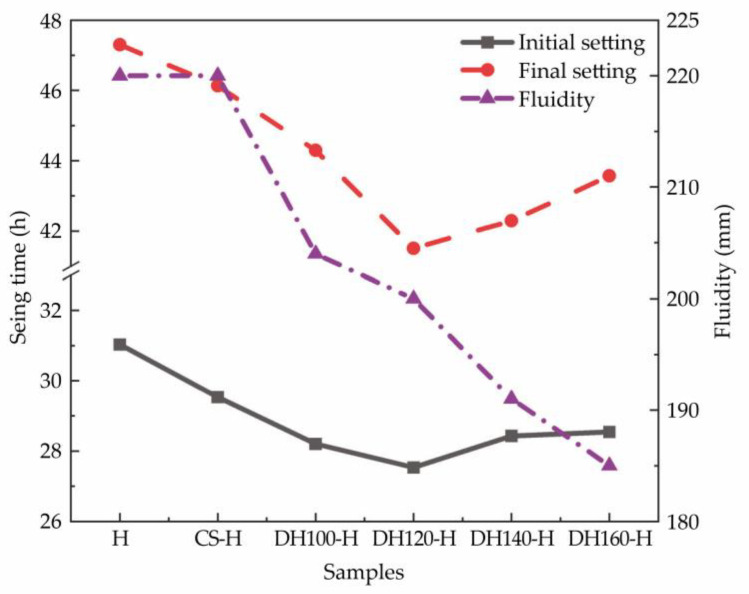
Setting times and fluidities of the high-phosphogypsum slag cement.

Figure 4 illustrates the setting time and fluidity characteristics of each experimental group: the control group (H group) exhibited initial and final setting times of 31.03 and 47.33 h, respectively. In addition, the seed modifier group showed reduced setting times of 29.53 h (initial) and 46.13 h (final), while the dehydration phase modifier group achieved the shortest setting times of 27.53 h (initial) and 41.47 h (final). These results clearly indicate that both the seed and dehydration phase modifiers effectively shortened the setting times of the phosphogypsum slag cement. Additionally, incorporating the dehydration phase modifier increased the paste viscosity by adsorbing water, thereby reducing the free water content and significantly lowering the fluidity.

### 4.2. Mechanical Performance

**Figure 5:**
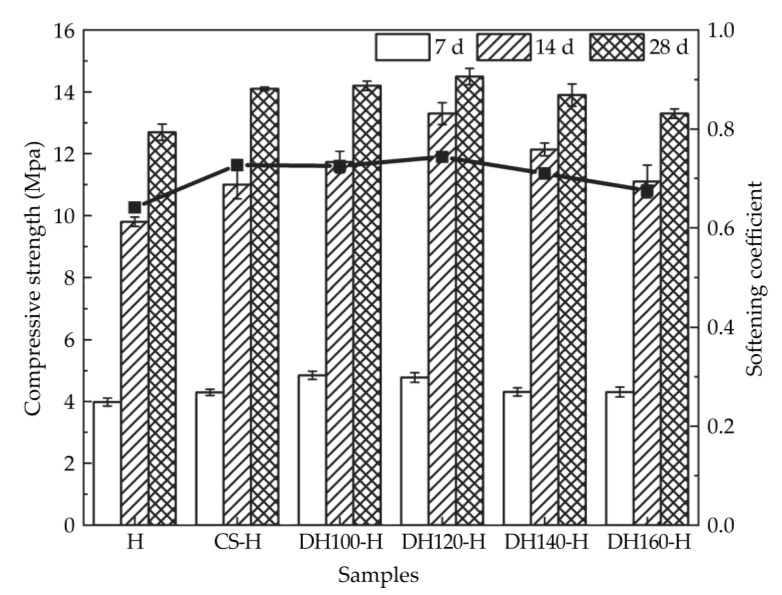
Compressive strengths and softening coefficients of the high-phosphogypsum slag cement.

Figure 5 presents the compressive strength and softening coefficient characteristics of the various samples at different curing ages. Relative to the control group (H), the CS-H group demonstrated 12.24 and 11.02% increases in its 14 and 28 d compressive strengths, respectively, although the DH120-H group exhibited the most significant improvements, with increases of 35.71 and 14.17%, respectively. However, increasing the heat treatment temperature of the dehydration phase had only a limited effect. For instance, the DH160-H group demonstrated only 13.27 and 4% increases in compressive strength compared to the control group for the 14 and 28 d measurements. In addition, both the seed and dehydration phase modifiers enhanced the early compressive strength. However, the water-absorbing properties of the dehydration phase modifier reduced the amount of free water, thereby providing more pronounced strength enhancement than the seed modifier. Nevertheless, the effectiveness of the dehydration phase modifier decreased with higher heat treatment temperatures, thereby highlighting the importance of temperature control.

**Figure 6:**
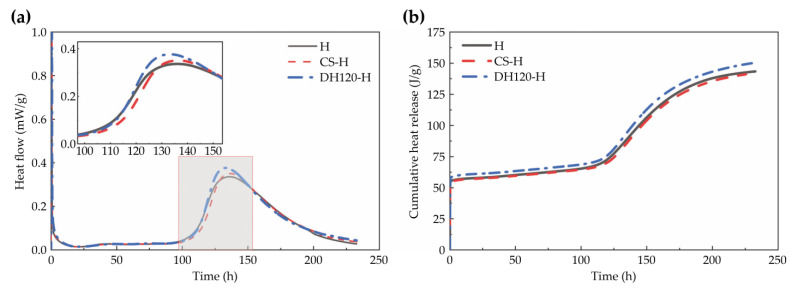
Heats of hydration for the high-phosphogypsum slag cement: (**a**) heat flow, and (**b**) cumulative heat release.

**Figure 7:**
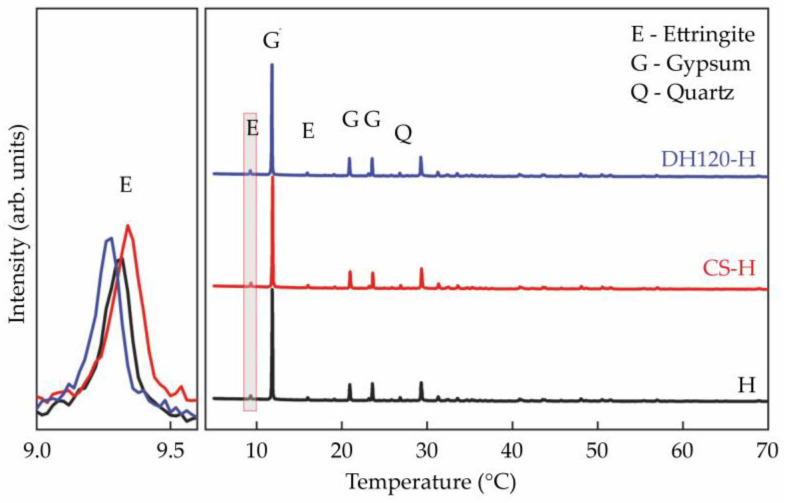
XRD patterns recorded for the 14 d hydration products.

The softening coefficients of both the DH120-H group (0.75) and CS-H group (0.72) were higher than those of the control group (0.64), with the DH120-H group demonstrating the best performance. This improvement is attributed to the modified pore structure of the specimens after the incorporation of modifiers, as illustrated in Figure 6 and Figure 7.

### 4.3. Hydration Process

**Figure 8:**
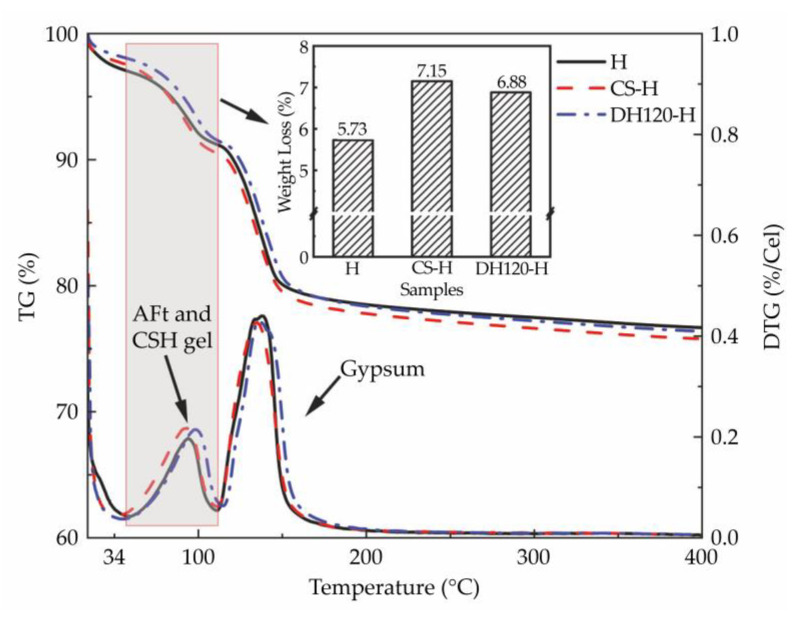
Thermograms of the 14 d hydration products.

As illustrated in Figure 8, the heat release curve of the high-content phosphogypsum slag cement paste exhibits two exothermic peaks, wherein the first peak corresponds to the dissolution of phosphogypsum and cement, releasing heat and increasing the Ca^2+^, SO_4_^2−^, and OH^−^ ion concentrations [23,24,25,26]. Upon hydration of the cement, the alkalinity increases, and the slag surface structure is disrupted, releasing Ca^2+^, Al^3+^, AlO_3_^3−^, and SO_4_^2−^, which react with OH^−^ to form the C-S-H gel and ettringite [27]; this represents the induction period. The second peak arises from continued slag dissolution and hydration product formation [28,29]. In this system, the low slag content results in a lower heat release rate. Consequently, the dehydration phase modifier group demonstrated a 12.12% increase in the rate of heat release corresponding to the second peak, with the peak occurring earlier; the seed modifier group showed a smaller enhancement of 4.40%. Therefore, the higher cumulative heat release exhibited by the dehydration phase modifier group confirms its role in promoting hydration.

### 4.4. Phase Analysis of the Hydration Products

**Figure 9:**
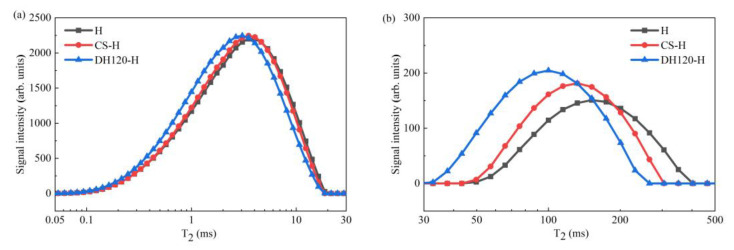
T_2_ spectra of the phosphogypsum cementitious material: (**a**) T_2_ spectrum between 0.05 and 30 ms, and (**b**) T_2_ spectrum between 30 and 600 ms.

**Figure 10:**
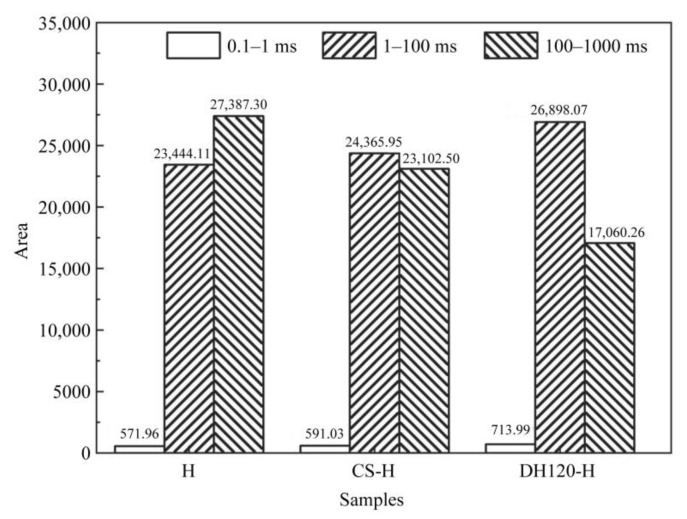
Integral areas of the T_2_ spectra recorded for the phosphogypsum cementitious materials.

The XRD and TG-DSC spectra of the 14 d specimens are illustrated in Figure 9 and Figure 10, wherein the main crystalline phases can be identified as dihydrate gypsum (CaSO_4_·2H_2_O, PDF#21-0816), ettringite (AFt, PDF#41-1451), and quartz (SiO_2_, PDF#78-2315). Ettringite, a principal hydration product, is characterized by its distinctive diffraction peak at 9.1° [30], and the intensity of this signal represents the degree of hydration. After normalization, the CS-H and DH120-H groups showed higher ettringite diffraction peak intensities and areas than the control group.

The thermogravimetric (TG) and derivative (DTG) curve presented in Figure 8 reveals two distinct endothermic events. The first thermal event (55–120 °C) is attributed to the decomposition of the C-S-H gel and ettringite components, while the second event (120–180 °C) corresponds to the thermal decomposition of the dihydrate and hemihydrate gypsum phases [31]. Quantitative analysis demonstrated that the mass loss associated with the first endothermic peak increased significantly upon modifier incorporation, with the seed modifier group exhibiting the most substantial enhancement (24.67%), followed by the dehydration phase modifier group (19.97%). These thermal analyses provide further evidence that both modifiers effectively accelerated the formation of hydration products, consistent with the phase composition analysis results obtained from the XRD measurements.

### 4.5. Microstructure Study

#### 4.5.1. LF-NMR

The pore architecture evolution in the 28 d-cured specimens was quantitatively assessed through LF-NMR the resulting transverse relaxation time (T_2_) distributions, and the corresponding integral areas are presented in Figure 6 and Figure 7. Three distinct pore regimes were identified based on the T_2_ values, namely, gel pores (0.01–1 ms, corresponding to interlayer water in C-S-H), capillary pores (1–100 ms, associated with hydration product interstices), and meso-macropores (100–1000 ms, representing structural defects and crack networks) [32]. Additionally, the bimodal T_2_ distributions revealed fundamental differences in the pore hierarchy between groups, wherein the control specimen (H group) exhibited predominant meso-macropore signals (integral area = 27,387.30) but limited gel pore development (integral area = 571.96). Upon the incorporation of the crystalline seed modifier, significant alterations in the pore structure of the cementitious system were observed. More specifically, the gel pore area increased to 591.03; the capillary pore area expanded to 24,365.95; and the meso-macropore area decreased to 23,102.50. Notably, the dehydrated phase modifier demonstrated enhanced regulatory effects, reaching the maximum values for both the gel water area (713.99) and the capillary pore area (26,989.07). This was accompanied by a substantial reduction in the meso-macropore area (17,060.26), the lowest observed among all experimental groups.

These results clearly indicate that modifier incorporation induced substantial pore structure refinement, particularly in the DH120-H specimen, which demonstrated optimal performance. Quantitative analysis showed 24.83 and 14.73% increases in the gel and capillary pore volumes, respectively, concurrent with a 37.71% reduction in the meso-macropore content. This threefold improvement mechanism, based on enhanced gel pore formation through accelerated C-S-H and ettringite nucleation, capillary pore densification via dehydration phase water absorption, and macropore elimination by seed-induced hydration product infilling, directly correlates with the observed enhancement in the compressive strength.

#### 4.5.2. SEM

**Figure 11:**
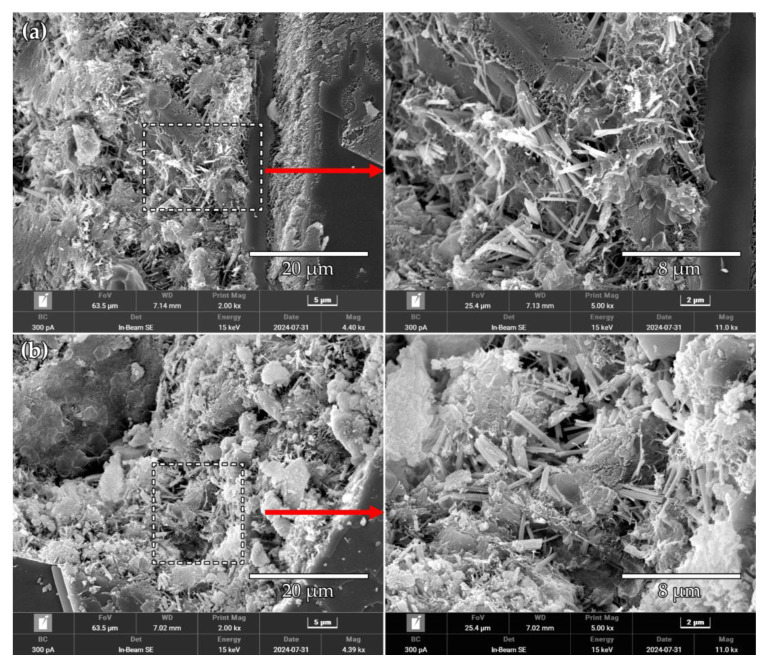
SEM images of the 28 d samples: (**a**) CS-H and (**b**) DH120-H samples.

FE-SEM observations were conducted on the fracture surfaces of the 28 d specimens to reveal the influences of the CS and DH modifiers on the microstructures of the high-content phosphogypsum cementitious materials. As indicated in Figure 11, unreacted phosphogypsum particles formed a three-dimensional skeletal structure, with the hydration products (i.e., ettringite and the C-S-H gel) acting as binding phases to fill the skeletal voids. In the DH group, needle-like ettringite crystals were interwoven to fill the pores while the C-S-H gel adhered to the ettringite surfaces, forming a dense matrix. In contrast, the CS group exhibited more exposed ettringite crystals without complete encapsulation by the C-S-H gel, resulting in a less dense pore structure compared to the DH group. This microstructural difference may account for the higher 28 d strength observed for the DH group compared to the CS group.

Based on the results and discussion presented above, the study identified 120 °C as the optimal calcination temperature, as the modifier prepared at this temperature significantly enhanced the hydration process of phosphogypsum-based cementitious materials, improved pore structure, and increased mechanical strength. Notably, the energy consumption and carbon emissions associated with the 4 h thermal activation process have yet to be quantified using a life cycle assessment. A comparative analysis of the carbon reduction benefits compared to those of conventional high-energy modifiers (e.g., silica fume) would be desirable; however, this approach would require further calculation of the net environmental benefit to validate any sustainability claims. More specifically, the net environmental gains from waste reuse must be rigorously weighed against the embodied energy of sustained heating (e.g., the electricity input required to maintain a temperature of 120 °C over 4 h on an industrial scale).

## 5. Conclusions

In this study, recycled cementitious modifiers, prepared via the mechanical activation and thermal treatment of phosphogypsum slag aggregate waste, were utilized to significantly improve the performance of high-content phosphogypsum cementitious systems. Notably, treating waste phosphogypsum-based aggregates generates both seed and dehydration phase modifiers, promoting the sustainable reuse of industrial byproducts. The main conclusions are as follows:

(1) The particle size of the DH modifier is smaller than that of the CS modifier. The phase composition of the DH modifier (primarily dehydrated gypsum and ettringite) is significantly influenced by the heat treatment temperature, with the hemihydrate gypsum content increasing as the temperature rises. Experimental optimization results indicate that 120 °C is the most effective heat treatment temperature, under which the generated components exhibit a well-balanced composition and demonstrate optimal mechanical performance.
(2) Both modifiers led to notable improvements in the compressive strength across all curing periods, with the most significant gains observed at 14 d. More specifically, the crystal seed-modified samples exhibited a 12.24% increase in strength, while the sample modified with the dehydration phase synthesized in the 120 °C group (DH120-H) achieved a more substantial 35.71% improvement, highlighting the superior performance of the dehydration phase modifier.
(3) The dehydration phase modifier demonstrated significant catalytic effects in terms of the hydration kinetics, with isothermal calorimetry revealing a 12.12% increase in the hydration heat release rate and a 4.9% elevation in the cumulative heat evolution. This acceleration reduced the initial setting time by 11.3% (i.e., from 31.0 to 27.5 h) and promoted the formation of hydration products, further enhancing the material performance.
(4) Both modifiers effectively refined the pore structure by increasing the proportion of gel pores (by 24.83%) and capillary pores (by 14.73%) while decreasing the mesopore and macropore areas (by 37.71%). This optimization resulted in a denser microstructure and improved the overall properties of the phosphogypsum cementitious material.

These results highlight the potential of utilizing phosphogypsum slag aggregate waste to produce high-performance modifiers, ultimately aiming to promote sustainable construction practices and waste management. Future research should focus on evaluating the long-term durability of these modifier–phosphogypsum cementitious materials, assessing the economic viability of large-scale production, and exploring the synergistic effects of combining these modifiers with other waste-derived materials to provide further performance and sustainability enhancements.

## Figures and Tables

**Table 1:** Chemical compositions of the raw materials (wt%).

Sample	Al_2_O_3_	SiO_2_	CaO	P_2_O_5_	SO_3_	Na_2_O	MgO	K_2_O	TiO_2_	Fe_2_O_3_	F	LOI
Phosphogypsum	0.795	6.714	28.785	0.837	38.747	0.174	0.849	0.32	0.10	0.467	0.969	21.171
OPC	5.126	22.24	61.189	0.20	2.612	0.20	1.913	0.883	0.239	3.078	——	1.992
GGBFS	16.132	33.952	34.91	0.061	2.913	0.96	6.865	0.458	0.942	1.152	——	0.885
RCMP	2.142	9.495	34.695	1.003	38.954	0.216	0.888	0.61	0.26	0.933	0.587	9.774

**Table 2:** Heat treatment conditions for preparation of the DH modifiers.

Group	Heat Treatment Temperature/°C
DH100	100
DH120	120
DH140	140
DH160	160

**Table 3:** Optimization of the modifier/high phosphogypsum slag cement mixing ratio.

Group	Modifier (wt%)		Phosphogypsum Cementitious Material (wt%)		
	Crystal Seed Modifier	Dehydrated Phase Modifier	Phosphogypsum	Slag	Cement
H	-	-	80	14	6
CS-H	5	-	80	14	6
DH100-H	-	5	80	14	6
DH120-H	-	5	80	14	6
DH140-H	-	5	80	14	6
DH160-H	-	5	80	14	6

**Table 4:** Particle size parameters of modifiers.

Sample	D_0.1_/μm	D_0.5_/μm	D_0.9_/μm	D _[4.3]_/μm	D _[3.2]_/μm
CS	2.096	15.296	54.054	22.533	5.534
DH100	1.99	12.924	52.226	20.883	5.164
DH120	2.047	13.453	53.347	21.39	5.265
DH160	1.989	13.675	49.852	20.482	5.2

## Data Availability

The original contributions presented in this study are included in the article. Further inquiries can be directed to the corresponding author.
